# Acknowledgments to reviewers of Zoological Research

**Published:** 2016-11-18

The editors of Zoological Research gratefully acknowledge the generous assistance of the following reviewers. We are thankful for the honest and invaluable work of all the referees during the past year, which has greatly contributed to ensuring the quality of Zoological Research.

**Table:** 

*Michael Braby* Australian National University, Australia	*Fang Zhou* Guangxi University, China
*Robert E. Schmidt* Bard College at Simon's Rock, USA	*Wei Liang* Hainan Normal University, China
*Yan-Yun Zhang* Beijing Normal University, China	*Jie Mei* Huazhong Agricultural University, China
*Joshua M. Hallas* California Academy of Sciences, USA	*Lu-Bin Jiang Jian-Hua Wang Chi-Yu Zhang* Institut Pasteur of Shanghai, Chinese Academy of Sciences, China
*Li Ding Jia-Tang Li Xiao-Mao Zeng* Chengdu Institute of Biology, Chinese Academy of Sciences, China	*Ke-Xin LI* Institute of Evolution, University of Haifa, Israel
*Lei Yang* College of Charleston, USA	*Huan-Zhang Liu Qiong-Ying Tang* Institute of Hydrobiology, Chinese Academy of Sciences, China
*Dai-Bin Zhong* College of Health Sciences, University of California, Irvine, USA	*Qiang Wei* Institute of Laboratory Animal Sciences, Chinese Academy of Medical Sciences, China
*Yun-Ke Wu* Cornell University, USA	*Xin-Zheng Li Jing Liu Feng You* Institute of Oceanology, Chinese Academy of Sciences, China
*Gerhard F. Weinbauer* Covance Laboratories GmbH, Muenster, Germany	*Yong-Hui Li* Institute of Psychology, Chinese Academy of Sciences, China
*Peng-Fei Fan* Dali University, China	*Qin-Qin Shi* Institute of Vertebrate Paleontology and Paleoanthropology, Chinese Academy of Sciences, China
*Fan Li* Fudan University, China	*Qing-Sheng Chi Wei Guo Yi-Bo Hu Xin-Hai Li Xuan Liu Chun-Guang Zhang Ya-Hui Zhao* Institute of Zoology, Chinese Academy of Sciences, China
*Harriet L. Robinson* GeoVax, Inc., USA	*Ce-Shi Chen Xiao-yong Chen Xue-Long Jiang Bing-Yu Mao Jun-Xing Yang Yong-Gang Yao Yun Zhang Yong-Tang Zheng* Kunming Institute of Zoology, Chinese Academy of Sciences, China
*Shi-Ping Gong Hui-Jian Hu* Guangdong Institute of Applied Biological Resources, China	*Yu-Yu Niu Tian-Rui Xu* Kunming University of Science and Technology, China
*Jian Yang* Guangxi Teachers Education University, China	*Nian-Hong Jang-Liaw* Taipei Zoo, China
*Bo Du* Lanzhou University, China	*Wai-Yee Chan* The Chinese University of Hong Kong, China
*Wen-Juan Wang* Nanchang University, China	*Hong-Shi Yu* The University of Melbourne, Australia
*Chang-Hu Lu* Nanjing Forestry University, China	*Luc Barbaro* University of Bordeaux, France
*Hong-Yu Liu* Nanjing Normal University, China	*Catherine E. Hart* University of Guadalajara, Mexico
*Eric Nelson* Nationwide Children's Hospital, USA	*Ana Ibarra-Macias* University of Miami, USA
*Jiong Chen Qing-Xi Han* Ningbo University, China	*Kevin Tang* University of Michigan-Flint, USA
*Craig V. Sullivan* North Carolina State University, USA	*Matthew Noakes* University of Pretoria, South Africa
*Zhong-Hua Liu* Northeast Agricultural University, China	*Mann Kyoon Shin* University of Ulsan, South Korea
*Bao-Guo Li* Northwest University, China	*Bo Zhang* Wuhan Institute Of Virology, Chinese Academy Of Sciences, China
*Kevin G Nyberg* Northwestern University, USA	*Peng Xu* Xiamen University, China
*Zi-Gui Chen Zhong-Xiao Hu* Ocean University of China, China	*Gui-Fen Li* Yulin Normal University, China
*Inga Mohrbeck Michael J. Raupach* Senckenberg am Meer, Germany	*Hong-Ning Zhou* Yunnan Institute of Parasitic Diseases, China
*Jia-Le Li Jin-Liang Zhao* Shanghai Ocean University, China	*Jian-Jun Gao Shu-Qun Liu Li Yu* Yunnan University, China
*Zhao-Bin Song* Sichuan University, China	
*Xiao-Yang Zhao* Southern Medical University, China	
*Xu Luo Wei Zhou* Southwest Forestry University, China	
*De-Yu Liao Jun-Hong Xia Hui Xu Peng Zhang Qiang Zhou*	
Sun Yat-sen University, China	

